# Integrative analysis of genomic and epigenetic regulation of endometrial cancer

**DOI:** 10.18632/aging.103202

**Published:** 2020-05-15

**Authors:** Qihang Zhong, Junpeng Fan, Honglei Chu, Mujia Pang, Junsheng Li, Yong Fan, Ping Liu, Congying Wu, Jie Qiao, Rong Li, Jing Hang

**Affiliations:** 1 Center for Reproductive Medicine, Department of Obstetrics and Gynecology, Peking University Third Hospital, Beijing 100191, China; 2 Beijing Key Laboratory of Reproductive Endocrinology and Assisted Reproduction, Beijing 100191, China; 3 Key Laboratory of Assisted Reproduction, Ministry of Education, Beijing 100191, China; 4 Institute of Systems Biomedicine, School of Basic Medical Sciences, Peking University Health Science Center, Peking University, Beijing 100191, China; 5 Department of Obstetrics and Gynecology, Tongji Hospital, Tongji Medical College, Huazhong University of Science and Technology, Wuhan 430000, China; 6 Key Laboratory for Major Obstetric Diseases of Guangdong Province, The Third Affiliated Hospital of Guangzhou Medical University, Guangzhou 510150, China; 7 Peking-Tsinghua Center for Life Sciences, Peking University, Beijing 100871, China

**Keywords:** proliferation, apoptosis, glycolysis, Wnt/β-catenin, gemcitabine

## Abstract

Endometrial carcinomas (EC) are characterized by high DNA copy numbers and DNA methylation aberrations. In this study, we sought to comprehensively explore the effect of these two factors on development and progression of EC by analyzing integrated genomic and epigenetic analysis to. We found high DNA copy number and DNA methylation abnormalities in EC, with 6308 copy-number variation genes (CNV-G) and 4376 methylation genes (MET-G). We used these CNV-G and MET-G to subcategorize the samples for prognostic analysis, and identified three molecular subtypes (iC1, iC2, iC3). Moreover, the subtypes exhibited different tumor immune microenvironment characteristics. A further analysis of their molecular characteristics revealed three potential prognostic markers (KIAA1324, nonexpresser of pathogenesis-related genes1 (NPR1) and idiopathic hypogonadotropic hypogonadism (IHH)). Notably, all three markers showed distinct CNV, DNA methylation, and gene expression profiles. Analysis of mutations among the three subtypes revealed that iC2 had fewer mutations than the other subtypes. Conversely, iC2 showed significantly higher CNV levels than other subtypes. This comprehensive analysis of genomic and epigenetic profiles identified three prognostic markers, therefore, provides new insights into the multi-layered pathology of EC. These can be utilized for accurate treatment of EC patients.

## INTRODUCTION

Endometrial carcinomas (EC) are epithelial malignant tumors that occur in the endometrium, and account for 20 to 30% of all tumors in the female reproductive system. The condition is one of the important causes of cancer-related deaths among women globally [1], ranked 4^th^ in female malignancies in developed countries, 7^th^ in the developing world [2], and second most common cancer among women in China. Although the 5-year survival rate of patients with early EC exceeds 90%, this rate in patients with distant metastases is below 20% [2]. Additionally, the prognosis of advanced, poorly differentiated or specific types of EC, is extremely poor necessitating identification of highly sensitive prognostic biomarkers to guide clinical management of patients with the disease.

Copy-number variations (CNVs), refers to DNA fragment copy number variations in the human genome, ranging from 1 KB to several Mb. These variations arise from single nucleotide polymorphisms (SNPs), deletions, insertions, replications of gene fragments, and variations of multiple sites [3]. Studies have reported that in a small number of patients with early EC, melatonin 2 (MSH2), MSH6, and PMS2, gene mismatch repair are correlated with high-risk germline mutations and have familial heritability. Some rare germline copy number deletions have been found in patients with EC, which interfere with genotypes, CpG islands, and sno/miRNAs, leading to deregulation of gene regulation and tumor development [4]. Studies have implicated glutathione thiol transferase T1 (GSTT1) gene copy number amplification in elevating the risk of EC, but not glutathione thiol transferase M1 (GSTM1) has been hypothesized to be a function of distinct substrate specificity of GSTT1 and GSTM1, since GSTT1 can generate subtypes of endometrial cells with genetic toxicity. Alterations in two or more numbers of CNVs, derived from GSTT1 genes, will increase the risk of EC [5]. In addition, inactivation or deletion of CCCTC-binding factor (CTCF) and zinc finger homeobox 3 gene (ZFHX3), encoded by tumor suppressor genes on chromosome 16q22 can also affect the occurrence of EC [6]. The risk of EC can, therefore, be effectively monitored using CNVs, and this enables early detection of specific genetic abnormalities.

Studies have shown that genetics and epigenetics overlap, and jointly regulate the occurrence and evolution of tumors. Due to abnormal methylation of promoters, the transcriptional level of tumor-related genes is increased in proliferating tumor cells and during tumor infiltration [7, 8]. Many tumor suppressor genes are mutated in type I EC. For example, O6-methylguanine DNA methyltransferase (MGMT) and adenomatous polyposis coli (APC) are inactivated by hypermethylation, resulting in tumorigenesis [9]. Studies have also shown that the RASSF1A promoter hypermethylation and KRas mutation-activated RAS pathways play an important role in the pathogenesis of EC [10].

In this study, we analyzed DNA copy numbers, and methylation as well as mRNA expression levels in a group of EC patients. We identified genes whose expression levels are regulated in genomic or epigenetic layers, and analyzed correlations among their expression. In addition, we used a multi-omics integration analysis to identify different molecular subtypes that are significantly associated with prognostic outcomes of EC. Furthermore, we performed a systematic analysis and identified new mutations that can be used as targets for precise treatment or biomarkers for subtype differentiation. Overall, our findings provide a basis for better understanding of the molecular pathogenesis of EC.

## RESULTS

**Figure 1:**
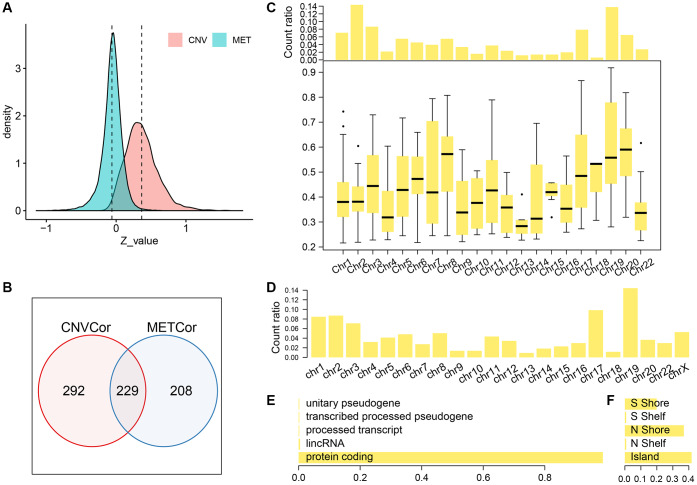
**Profiles of CNV-G and MET-G features.** (**A**) z-value distribution of CNV-G and MET-G. (**B**) The overlap between CNV-G and MET-G. (**C**) Chromosome distribution (top panel) and correlation (bottom panel) of CNV-G. (**D**) Chromosome distribution of MET-G. (**E**) MET-G gene type. (**F**) The proportion of MET sites.

We identified a total of 6308 copy-number variation genes (CNV-G) and 4376 methylation genes (MET-G). Analysis of the z-value distribution, indicated that the correlation between CNV-G and the corresponding gene expression profiles clearly shifted to the right, while that between MET-G and the corresponding gene expression profiles significantly shifted to the left **(**CNV-G skewness = 0.83226, MET-G skewness = -0.79108) (Figure 1A). A further analysis, using the Fisher's z-transformation at 95% confidence interval, revealed a positive correlation gene in signature for DNA copy numbers (CNV-G, n=521) and a negative correlation gene signature for DNA methylation (MET-G, n=437). CNV-G and MET-G showed an overlap of only 229 genes, which suggested that dysregulation of CNV-G and MET-G transcription was mutually exclusive (ratio: 43.9/52.4%) (Figure1B). CNV-G and MET-G genes showed regional genomic preferences and were mostly located on chromosome 19 (Figure 1C, 1D). Additionally, we found MET-G, which are mainly a protein-coding gene (Figure 1E), and MET sites were mostly in the CpG island, N, and S Shore intervals (Figure 1F).

### Molecular subtypes based on CNV-G and MET-G genes

**Figure 2:**
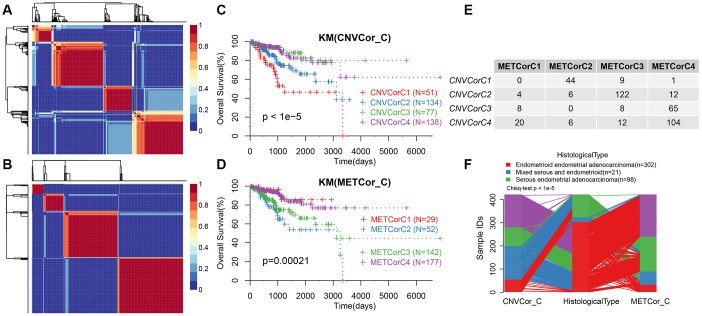
**Molecular subtypes based on CNV-G and MET-G genes.** (**A**) NMF-based clustering of CNV-G. (**B**) NMF-based clustering of MET-G. (**C**) KM survival curve of CNV-G subtype. (**D**) KM survival curve of MET-G subtype. (**E**) The overlap between the CNV-G subtype and the MET-G subtype. (**F**) The overlap between the CNV-G subtype, the MET-G subtype and the histological subtype.

Next, we determined whether the expression profiles of CNV-G and MET-G genes could predict prognosis. Cluster analysis showed an optimal clustering number of 4 for both CNV-G and MET-G (Figure 2A and 2B). Kaplan-Meier (KM) plots, for overall survival (OS), revealed significant differences in prognostic outcomes between the groups (Figure 2C), with marked differences observed in the MET-G subclass (Figure 2D). In addition, there was a significant overlap among the four subclasses of both CNV-G and MET-G (Figure 2E, 2F).

### CNV, MET, and EXP datasets were integrated for cluster analysis

**Figure 3:**
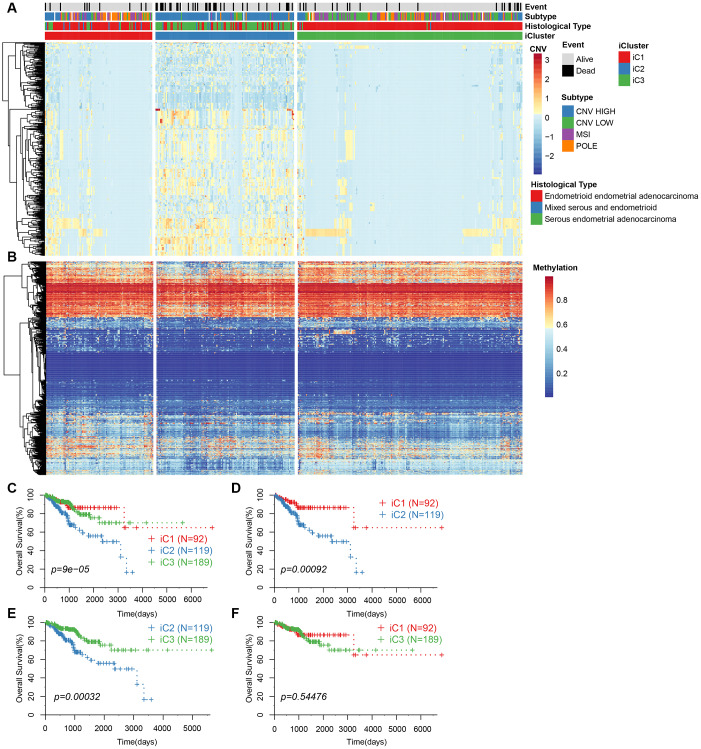
(**A**) CNV levels of subtype CNV-G identified by iCluster. (**B**) Methylation levels of MET-G subtype identified by iCluster. (**C**) KM curve for the subtypes identified by iCluster. (**D**) KM curve for iC1 and iC2 subtypes. (**E**) KM curve for iC2 and iC3 subtypes. (**F**) KM curve for the iC1 and iC3 subtypes.

We repeated the clustering 20 times in K=2 (class 3) and K=3 (class 4) in order to optimize clusters created by iCluster. The results indicated a more stable clustering in rank=2 (class 3) than rank=3 (class 4). We finally concluded that the iCluster was aggregated into three subclasses: iC1 (95 samples), iC2 (128 samples), and iC3 (198 samples). Based on the CNV level distribution of iCluster, we found a higher CNV in iC2 compared to iC1 and iC3 (Figure 3A), although their methylation levels were comparable between the groups (Figure 3B). A further comparison of iCluster and existing EC subtypes revealed that iC2 mainly corresponded to CNV high subtypes, whereas CNV low and MSI subtype samples were mainly concentrated in iC3 (Figure 3A). KM survival analysis indicated significant differences in OS between the three groups (Figure 3C). Further comparisons among the three groups revealed significantly different prognosis among iC1, iC3 and iC2 subtypes (Figure 3D, 3E). However, there was no significant difference in prognosis between iC1 and iC3 subtypes (Figure 3F). Moreover, progression-free survival (PFS) was significantly different among the three subtypes (Supplementary Figure 1).

### Abnormality in DNA copy number is consistent with methylation abnormality

**Figure 4:**
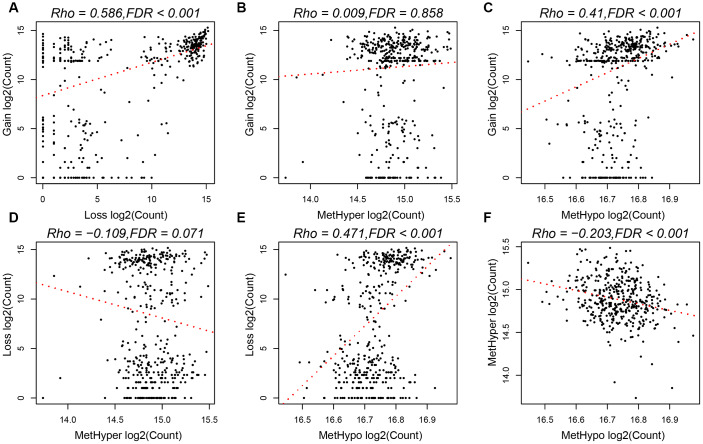
(**A**) Frequency distribution of CNV Gain and Loss. (**B**) Frequency distribution of CNV Gain and MetHyper. (**C**) Frequency distribution of CNV Gain and MetHypo. (**D**) Frequency distribution of CNV Loss and MetHyper. (**E**) Frequency distribution of CNV Loss and MetHypo. (**F**) Frequency distribution of MetHyper and MetHypo.

To study the relationship between CNV and MET abnormalities, we defined the *β* value of CNV > 0.3 as CNV Gain; *β* value < -0.3 as Loss; *β* value of MET > 0.8 as MetHyper (hypermethylation); and *β* value < 0.2 denoted as MetHypo (demethylation). We counted the numbers of CNV Gain, Loss, MetHyper and MetHypo for each sample and found a significant correlation between Gain, Loss and MetHypo (Figure 4A, 4C), not with MetHyper (Figure 4B). Additionally, we did not record a significant correlation between Loss and MetHyper (Figure 4D), although both were significantly correlated with MetHypo (Figure 4E). MetHyper and MetHypo showed a strong negative correlation (Figure 4F).

### Characteristics of the tumor microenvironment among the molecular subtypes

**Figure 5:**
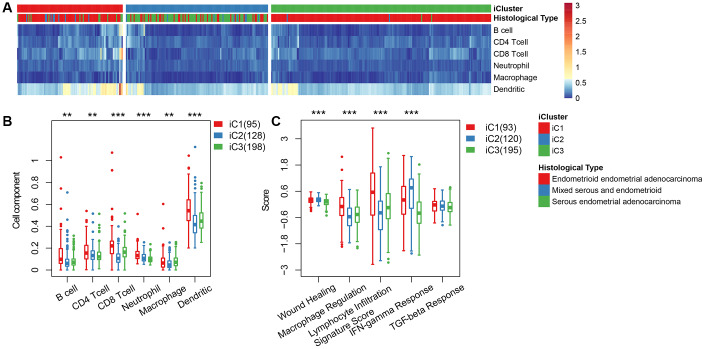
(**A**) Immune cell scores obtained from all samples. (**B**) A comparison of all immune cell scores among the three subtypes of iCluster. (**C**) A comparison of 5 immune signatures scores.

We categorized EC into three subgroups according to multi-group data, then compared the differences in clinical characteristics of iC subtypes in stage, grade, age, and BMI (body mass index). Results revealed significant differences in the distribution of iC subtypes among samples with different clinical characteristics (Table 1). High-grade and advanced samples were more likely to be distributed in the worst prognostic iC2 subtype (Supplementary Figure 2). We used tumor immune estimation resource (TIMER) to compare immune scores across the three subtypes, and found that six immune cell scores were lower in iC2 subtype and had the worst prognosis compared to the other subtypes (Figure 5A, 5B), indicating that the iC2 subtype may represent an immunosuppressive state. Comparative analysis further showed a significantly lower macrophage regulation and lymphocyte infiltration score in the iC2 subtype relative to the other subtypes, whereas the wound healing and inflammation (IFN-gamma response)-related score was significantly higher in iC2 than other subtypes (Figure 5C). This further suggested that immune status may affect the prognosis of EC.

**Table 1:** Comparison of clinical features between EC subtypes.

**Clinical Features**	**Total**	**iC1**	**iC2**	**iC3**	**p value**
Event					0.00076
Alive	351	84	92	175	
Dead	68	10	35	23	
NA	2	1	1	0	
Stage					<0.001
I	255	65	49	141	
II	42	7	16	19	
III	100	18	49	33	
IV	24	5	14	5	
Grade					<0.001
G1	60	7	2	51	
G2	87	10	9	68	
G3	263	75	109	79	
G4	11	3	8		
New Event Type					0.0011
Distant Metastasis	12	3	3	6	
Locoregional Recurrence	25	2	15	8	
New Primary Tumor	5	4	1	0	
Primary	361	83	99	179	
Un	18	3	10	5	
Age					<0.001
31~50	37	16	0	21	
50~60	100	21	17	62	
60~70	153	33	56	64	
70~80	92	18	37	37	
80~90	39	7	18	14	
Body Mass Index					0.00036
0~26.22	118	37	45	36	
26.22~32.24	106	22	34	50	
32.24~38.69	94	13	29	52	
38.69~214	103	23	20	60	

### Molecular characteristics of the subtypes

**Figure 6:**
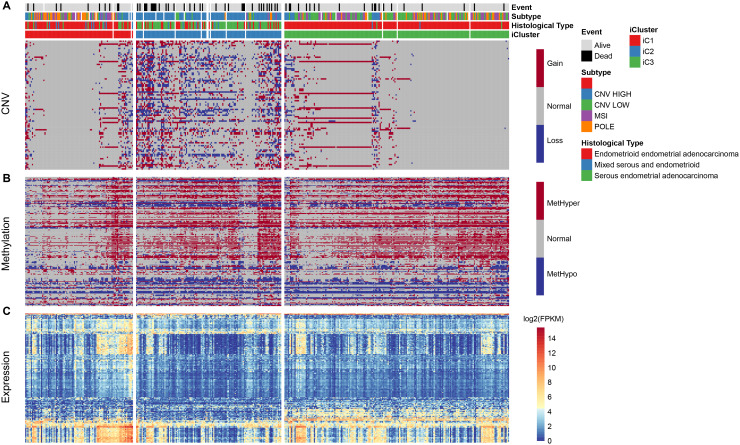
(**A**) Distribution pattern for CNV in iCluster. (**B**) Distribution for methylation level in iCluster. (**C**) Heatmap of differentially expressed genes in iCluster subtypes.

**Figure 7:**
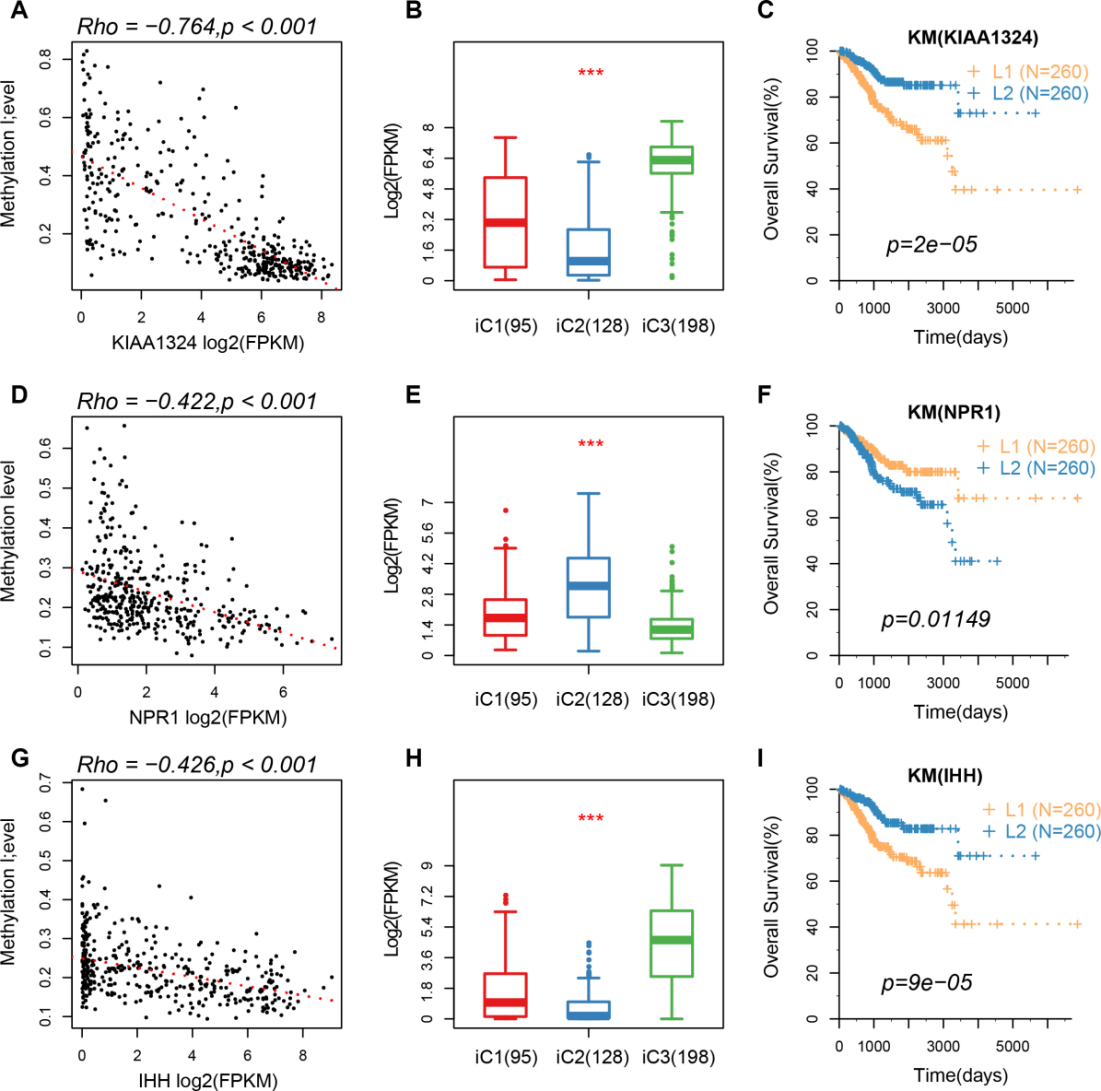
(**A**–**C**) A correlation of methylation of KIAA1324 gene with its expression, expression of iC subtypes, and the KM curve of the high/low expression groups. (**D**–**F**) A correlation of methylation of NPR1 gene with its expression, expression of iC subtypes, and the KM curve of the high/low expression groups. (**G**–**I**) The relationship between methylation of IHH gene with its expression, expression of iC subtypes, and the KM curve of the high/low expression group.

Based on the results from iCluster, we compared the differentially expressed genes (DEGs) between iC1/iC3 and iC2. We identified a total of 207 DEGs in the three groups, after removing the low expression levels. Gene ontology (GO) analysis indicated a significant enrichment of terms related to immune regulation, such as leukocyte migration and adaptive immune response. The CNV frequency of 207 DEGs in iC2 was significantly higher than that in iC1 and iC3, suggesting that CNV influenced the prognosis of EC (mean CNV: 8005/28579/5899) (Figure 6A). However, no significant differences were observed in methylation levels between the molecular subtypes (mean methylation: 134682/143148/140185) (Figure 6B). A correlation between expression level, methylation and CNV, revealed a high expression of DEGs in demethylated samples (Figure 6C), but this was not observed in CNV. This indicated that the effect of methylation on the expression of DEGs was stronger than the effect of CNV on the expression of DEGs. Univariate survival analysis identified 24 genes that were significantly associated with prognosis. We also analyzed expression of three genes: KIAA1324, nonexpresser of pathogenesis-related genes1 (NPR1) and idiopathic hypogonadotropic hypogonadism (IHH), and the relationship between methylation and CNV. We also found a significant negative correlation between expression of these genes and their methylation status, but not in the CNV (Figure 7). Studies have shown that KIAA1324 is activated by estrogenase, which suggests that estrogenase may play a role in the occurrence of EC.

### Mutation spectrum of molecular subtypes

**Figure 8:**
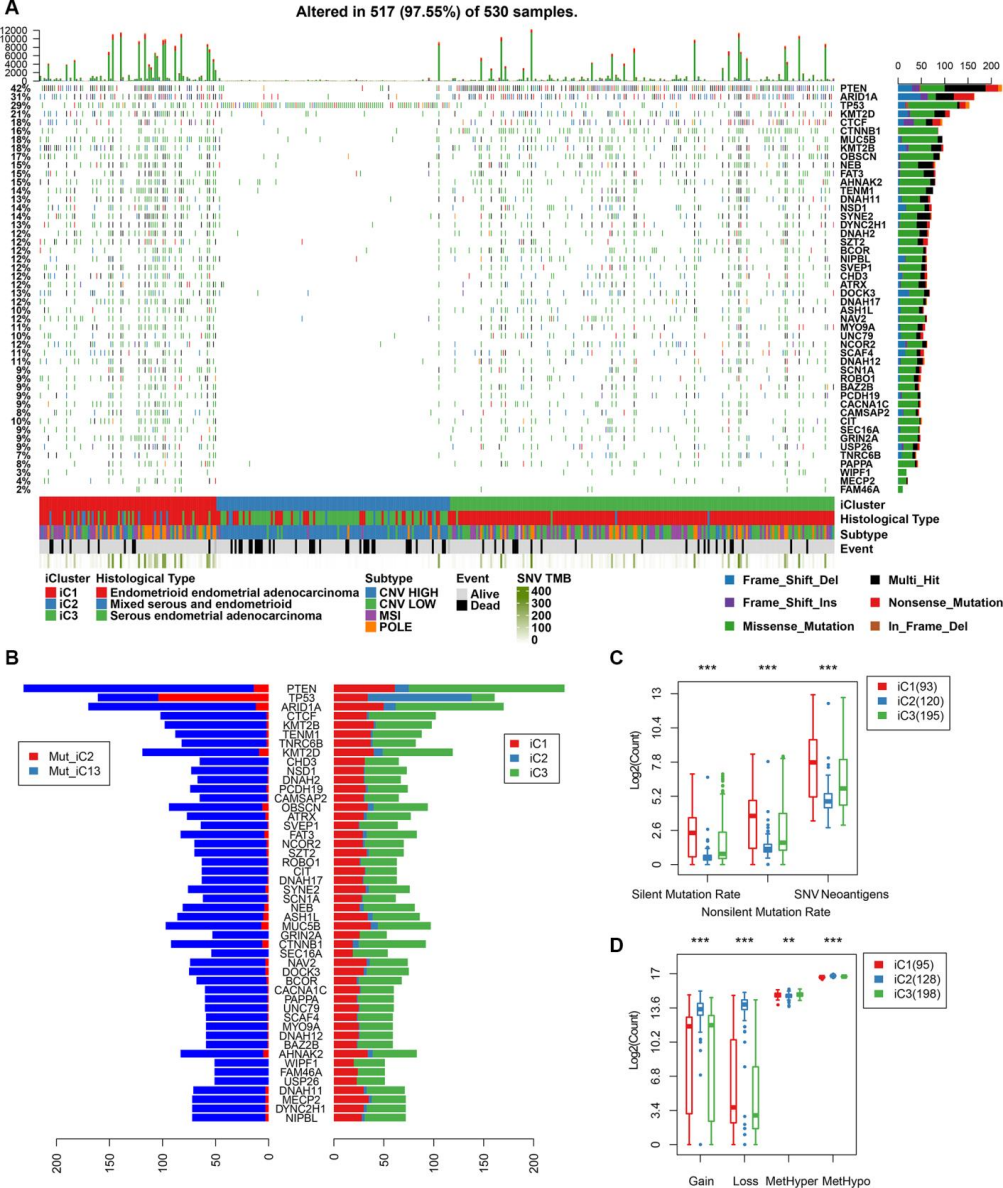
(**A**) Profiles of significant mutations in 48 genes across iC subtypes. (**B**) Distribution pattern of mutation number of the 48 genes with significant mutations. (**C**) Distribution of silent/nonsilent and neoantigens among iC subtypes. (**D**) Distribution of CNV Gain/Loss and methylated MetHyper/MetHypo among iC subtypes.

We further mapped the mutation spectrum of various molecular subtypes to identify differentially expressed genes in iC subtypes. Using the Fisher’s test (with FDR < 0.001), we obtained a total of 48 genes. Mutation spectrum analysis showed a significantly lower mutation frequency of PTEN, ARID1A, CTNNB1 in the iC1/iC3 subtype with better prognosis than that in the iC2 subtype (FDR < 0.0001). Moreover, the mutation frequency was significantly higher in subtype iC2 than in iC1/iC3 (Figure 8A). Overall, there were fewer silent/nonsilent mutations and neoantigens in iC2 than in iC1/iC3 (Figure 8B). However, the number of CNVs in iC2 was significantly higher than that of iC1/iC3 (Figure 8C), suggesting that the effect of gene copy number variation on prognosis was stronger than that of genomic mutations. Additionally, methylated MetHyper/MetHypo levels varied significantly among the molecular subtypes (Figure 8D).

## DISCUSSION

In the present study, we defined the genome and epigenome of CNV-G and MET-G genes by integrating multiple sets of genomic and epigenetic data. Our results revealed genes that successfully identified EC subtypes and exhibited good prognostic value. The results further indicated that ECs with higher CNV-G aberrations harbored corresponding high MET-G aberrations, suggesting that patients with frequent DNA copy number aberrations are more prone to DNA methylation aberrations. Notably, analysis of the classification based on CNV-G and MET-G, revealed novel molecular features that have potential to be biomarkers for identifying EC. Comparison of mutant profiles among the molecular subtypes revealed distinct mutation rates of BAP1 and CTNNB1.

Tumor-infiltrating lymphocytes are activated via diverse mechanisms and cytokines, all of which elicit multiple immune responses, directly or indirectly affecting other components in the tumor microenvironment thereby modulating development of tumors. Numerous studies have implicated TILs in clinical prognosis of cancer patients. For instance, Shia et al. [11] reported that high levels of TILs and peritumoral lymphocyte infiltration could predict microsatellite instability (MSI) in EC with 85 and 46% sensitivity and specificity, respectively. Similarly, Asaka et al. [12] reported that EC patients with mismatch repair deficiency show higher levels of CD8^+^ T cells, Tregs, and PD-1^+^ immune cells, while Workel et al. [13] found a correlation between elevated CD8^+^ PD-1^+^ lymphocytes and better EC prognosis. Other studies have shown that high numbers of Treg cells correlate with poor prognosis of patients with EC [14]. T.Bosse et al. identified and studied four molecular subgroups, including POLE ultramutated (POLEmut), mismatch repair-deficient (MMRd), p53 mutant (p53abn) and NSMP (non-specific molecular profile) for EC [15]. However, a comprehensive analysis of larger data sets is required to confirm the prognostic value of TILs in EC. In the current study, the immune cell score of iC2 subtype was significantly lower than that of other subtypes. Moreover, macrophage regulation and lymphocyte infiltration scores of iC2 subtype were significantly lower than those of other subtypes, while the wound healing and IFN-gamma response scores were significantly higher in iC2 compared to the other subtypes. This indicated that activity of the immune system has a profound prognostic role in EC.

Furthermore, we found a significant association between three genes (KIAA1324, NPR1, and IHH) with prognosis of EC. The KIAA1324 gene is a new estrogen-inducing gene that is differentially regulated in endometrial and non-EC [16]. Additionally, expression of NRP1 protein is significantly up-regulated in gastric cancer tissues and cell lines [17]. However, the role of this protein in regulation of EC is not known. Wang [18] reported a six-gene signature with prognostic value for patients with EC, which included IHH. The results of the present study show a strong negative correlation between these genes and methylation processes, suggesting that their expression may be affected by epigenetic regulation. These genes are, therefore, potential prognostic markers for patients with EC.

Although the relationship between epigenetic and genomic variation was successfully established using bioinformatics tools, this study had some limitations: 1) the data lacked some clinical follow-up information, thus other factors such as health status of the patient were not considered in the prediction of clinical outcomes; 2) the data analyzed here was obtained via bioinformatics analysis, and hence may be inadequate. 3) This study is based on multidimensional omics data. In the process of processing, the reproducibility of data tends to decrease due to the different processing methods of omics data of samples. Therefore, further genetic experimental studies involving larger sample sizes are needed to validate our findings.

In summary, this study successfully evaluated the possible pathogenesis of EC through multi-omics data analysis of genomics, epigenetics and transcriptomics, and demonstrated that CNV and methylation play an important role in EC. In addition, three clinically-relevant molecular EC subtypes and three critical biomarkers of the disease were identified. These novel mechanisms and clinical classifications will be vital in development of accurate and targeted therapies for patients with EC.

## MATERIALS AND METHODS

### Data collection

We downloaded recent clinical follow-up information from The Cancer Genome Atlas (TCGA) (https://portal.gdc.cancer.gov/) using the TCGA GDC API (https://gdc.cancer.gov/developers/gdc-application-programming-interface-api) at 2019.01.24. CNV, Methylation, RNA-seq data and the SNP data processed by the mutect software were also downloaded. The RNA-seq data also includes the UCEC count for subsequent group differential expression analysis. A total of 161 samples of all three sets of data used in subsequent analyses.

### Data preprocessing

The following processing procedures were performed on the CNV, Methylation, RNA-seq, and SNV datasets in the EC samples from the TCGA database:

### CNV data preprocessing

The CNV intervals were merged using the criteria shown below:

1. 50% regional overlap between two intervals was considered as the same interval.
2. The number of coverage probes <5 intervals were removed.
3. The CNV interval was mapped to the corresponding gene using the GRh38 version of 22.
4. Multiple CNV regions in one gene region were combined into one, and the combined CNV value was averaged.

### Preprocessing of methylation data

1. Missing sites in more than 70% of the samples were removed.
2. KNN (k-nearest Neighbour) algorithm was applied to fill in missing values.
3. Probes 2 kb upstream and 200 bp downstream of the TSS intervals were retained by the annotated version of gencode.v22 and mapped to the corresponding genes.

### Preprocessing RNA-seq data

1. Lowly expressed genes (samples with FPKM of 0 accounting for <0.5 of all samples) were removed.

### SNV data preprocessing

1. Mutations in the intron interval were removed.
2. Mutations annotated as silence were removed.

### Identification of CNV-G and MET-G

We calculated Spearman correlation coefficients for each gene corresponding to CNV and expression profile (RNA-seq) and methylation and expression profile, respectively. These coefficients were then converted into z-value using the formula ln(1+r)/(1-r). Genes with p < 1e-5, which were tested for correlation coefficients, constituted a CNV-G and a MET-G.

### Identification of molecular subtypes of CNV-G and the MET-G

Nonnegative matrix factorization (NMF) is an unsupervised clustering method widely used to identify molecular subtypes of tumors based on genomics [19, 20]. To further explore the association between CNV-G and MET-G expressions with clinical phenotypes, samples were clustered using the NMF method based on the expression profiles of the CNV-G and MET-G gene sets. Briefly, we selected the standard "brunet", using the NMF method 50 iterations. The number of clusters k was set between 2 to 10, and the average profile width of the common member matrix calculated using the NMF package implemented in R [21], with the minimum member of each subclass was set to 10.

### Identification of molecular subtypes

We employed the R package ‘iCluster’ [13], to perform multi-group data integration cluster analyses and integrate the copy number variation (CNV) data of the CNV-G gene, methylation data (MET) of the MET-G gene, as well as the expression profile data (EXP) of the genes in CNV-G and MET-G. Subsequently, 20 iterations and10 lambda sample points between 0-1 were used for optimal lambda value screening to identity optimal CNV, MET, and EXP data weight values (lambda values). Considering the number of molecular subtypes identified by CNV-G and MET-G, we chose 2-4 as the number of clustering K.

### Relationship between molecular subtypes and tumor microenvironment

Tumor immune estimation resource (TIMER) is a platform used for systematic assessment of the clinical impact of different immune cells in various types of cancer [22]. This method was used to estimate the abundance of six immune cell types, namely: B, CD4 T, CD8 T, and neutral cells, macrophages and dendritic cells. The abundance of those cells in the tumor microenvironments were analyzed in different molecular subtypes.

### Analysis of genetic differences in molecular subtypes

To examine differences in gene expression among the molecular subtypes, we employed DESeq2 [23] tool using 2-fold differences and FDR < 0.05 as thresholds for identifying differentially expressed genes between molecular subtypes.

### Relationship between molecular subtypes and tumor genomic variation

To assess the differences in genomic variation between molecular subtypes, we downloaded SNP data from TCGA, then removed introns and silent mutations. We then used the Fisher's exact test to compare differences in mutations between the two samples. A threshold of p<0.05 was used to identify mutated genes.

### Functional enrichment analysis

Gene Ontology (GO) and Kyoto Encyclopedia of Genes and Genomes (KEGG) pathway enrichment analyses were performed using the R package cluster profiler 28 for genes. We then identified over-represented GO terms in three categories namely; biological process, molecular function, and cellular component. For these analyses, a FDR < 0.05 was considered for the determination of statistical significance.

### Statistical analysis

Kaplan-Meier was used to visualize the differences in subtype prognosis, while univariate survival analysis was performed to estimate overall survival. The log-rank test was used to test prognostic differences at a significance of *p* < 0.05. All the analyses were performed in R software version 3.4.3.

## Supplementary Material

Supplementary Figures
