# Supplementary material for: Integrative analysis of genomic and epigenetic regulation of endometrial cancer
Source: Aging (Albany NY). 2020 May 15;12(10):9260–74. doi: 10.18632/aging.103202 (PMC7288931; doi:10.18632/aging.103202)
Supplement: Supplementary Figures [file aging-12-103202-s001..pdf]

SUPPLEMENTARY FIGURES

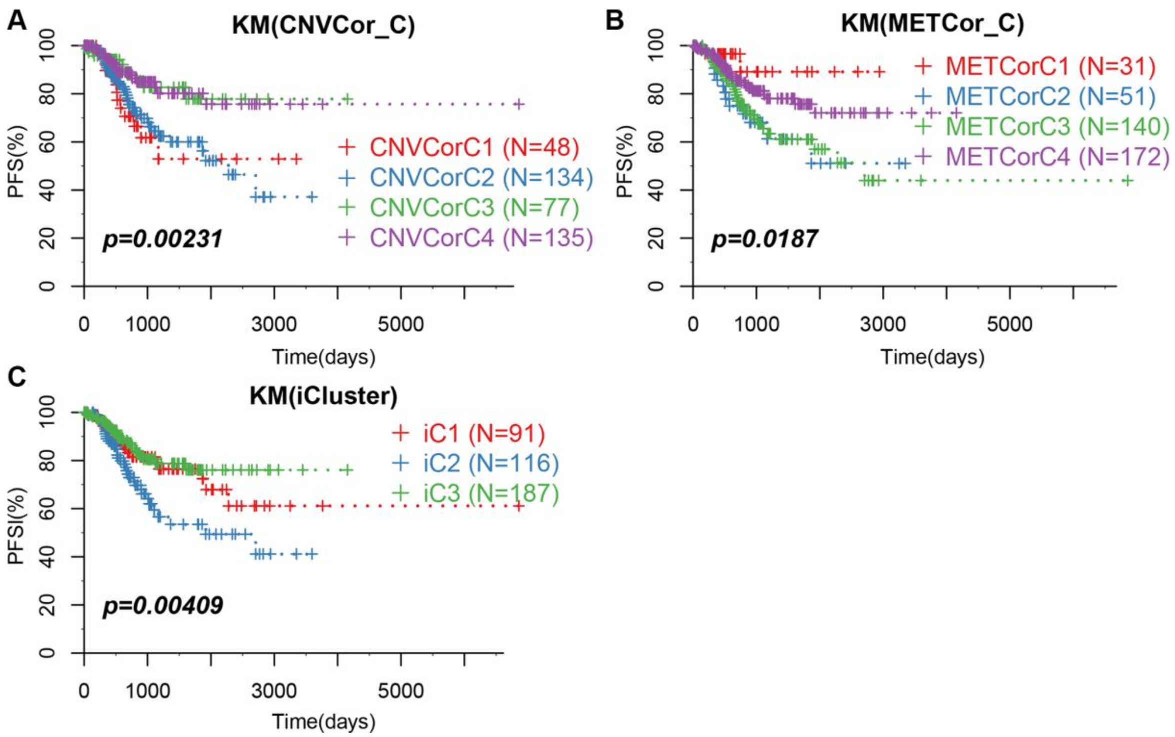

Supplementary Figure 1. (**A**) Progression-free survival among CNVCorC1, CNVCorC2, CNVCorC3 and CNVCorC4. (**B**) Progression-free survival among METCorC1, METCorC2, METCorC3 and METCorC4. (**C**) Progression-free survival among iC1, iC2 and iC3.

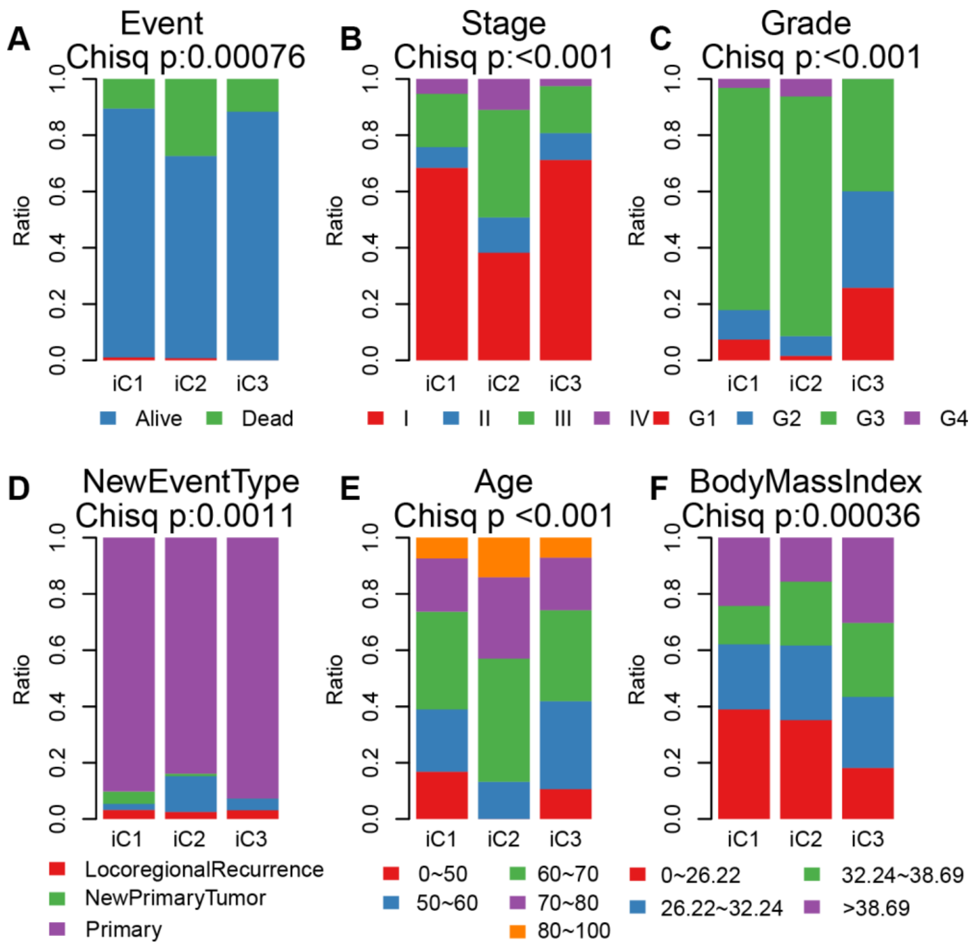

Supplementary Figure 2. (**A**) The distribution of Event in three iC types. (**B**) The distribution of Stage in three iC types. (**C**) The distribution of Grade in three iC types. (**D**) The distribution of NewEventType in three iC types. (**E**) The distribution of Age in three iC types. (**F**) The distribution of BodyMassIdex in three iC types.
